# Introducing and evaluating a knowledge transfer approach to support problem solving in and around protected areas

**DOI:** 10.1007/s13280-018-1048-5

**Published:** 2018-03-30

**Authors:** Brady J. Mattsson, Marie Fischborn, Mark Brunson, Harald Vacik

**Affiliations:** 1 Institute of Silviculture, Institute of Wildlife Biology & Game Management, University of Natural Resources and Life Sciences (BOKU), Gregor-Mendel-Straße 33, 1180 Vienna, Austria; 2 Global Protected Areas Programme, International Union for Conservation of Nature (IUCN), Rue Mauverney 28, 1196 Gland, Switzerland; 3 Department of Environment and Society, Utah State University, 5215 Old Main Hill, Logan, UT 84322-5215 USA

**Keywords:** Nature conservation, PANORAMA Solutions for a Healthy Planet, Peer learning, Protected area management, Sustainable development, Web platform

## Abstract

Protected areas (PAs) can generate many benefits inside and outside their borders, and achieving objectives for diverse stakeholders raises many challenges. There are many examples of successful PA management around the globe, although a systematic and comprehensive approach to developing and sharing these solutions has been lacking. We present “solutioning” as a structured process of peer-learning, which can inform management strategies in and around protected areas. We explain how the PANORAMA—Solutions for a Healthy Planet initiative has put solutioning into practice through an interactive community and web portal to learn about protected area solutions around the globe. Unlike other web platforms and initiatives reviewed, PANORAMA facilitates adaptation of solution elements (i.e., building blocks) for novel implementation. Supported by theories of resilience and peer-learning, PANORAMA appears to have potential to promote efficiency and equitable benefits for PAs and associated stakeholders focused on nature conservation and sustainable development, although further research is needed to assess whether this learning leads to better solutions or more effective PA management.

**Electronic supplementary material:**

The online version of this article (10.1007/s13280-018-1048-5) contains supplementary material, which is available to authorized users.

## Introduction

Protected areas (PAs) around the world cover 15% of terrestrial and freshwater areas and 4% of marine areas within national jurisdiction (Bhola et al. 2016), playing an essential role for maintaining biodiversity, ecosystem integrity, and the diverse services and cultural values these landscapes and ecosystems provide to society (Watson et al. 2014). Objectives of individual protected areas (PAs) can vary greatly (Dudley 2008), and through diverse management practices PAs generate many benefits including climate change mitigation and adaptation, regulating erosion and flooding, protecting watersheds for water quality, providing habitat for wild species, protecting sacred grounds for indigenous peoples and other communities, and supporting regional development (Watson et al. 2014; Stolton et al. 2015). Through participatory approaches, stakeholder groups engaged in PA management (e.g., PA managers, local communities, and non-government organizations; henceforth PA stakeholders) have the potential to affect conservation and natural resource management beyond borders of PAs (Wells and McShane 2004; Schick et al. 2017). As such, PAs can serve as nodes or hubs that enable flows of ecosystem services in green infrastructure networks at broader scales (Benedict and McMahon 2012; Mattsson and Vacik 2018).

Enabling equitable provision of ecosystem services and associated benefits for diverse stakeholders over the long term (henceforth, socioecological sustainability; Broto 2013) is an ambition drawn from global policy documents by the United Nations (i.e., Sustainable Development Goals; UN General Assembly 2015) and the International Union for Conservation of Nature (IUCN; Dudley 2008) and is relevant for many protected areas around the planet. Achieving objectives across the socioecological spectrum (e.g., species protection and recreation opportunity) even at local to regional scales raises many challenges for management of individual PAs. Among others, these challenges include gaps in knowledge to develop and implement effective management plans that balance competing objectives, and insufficient communication and engagement among stakeholders (Ferraro and Hanauer 2015). In addition to these internal challenges, external pressures well beyond the control of PA stakeholders are influencing decision-making, including political shifts, pressures for economic development, PADDD (PA downgrading, downsizing, degazettement), climate change impacts, and invasive species (Watson et al. 2014; Watson et al. 2016). Efficient solutions are needed urgently to overcome these challenges to ensure the long-term benefits of PAs for nature and society.

Many knowledge resources exist to help PA stakeholders address the key challenges, and they exist primarily in the form of general guides, training and tutorials, and as individually published case studies (Table 1). Case studies that describe the implementation of a particular approach in a local or regional context are particularly valuable, because they highlight the challenges and benefits associated with particular solutions (i.e., successfully implemented management strategies) in a specific area. PA managers in Europe have expressed interest in sharing more detailed descriptions of such case studies that have been implemented for individual PAs or PA networks, in a format that they can easily understand and interpret. This presents a challenge, as each solution is tailored to the focal PA(s), and solutions are reported in various formats that may preclude easy sharing and consistent interpretation by other PA stakeholders.

**Table 1:** Examples of knowledge resources highlighting best practices for management of individual protected areas around the globe

Author(s), year, title
General guides and publications
IUCN^a^ (2016), Protected areas: best practice guidelines.
Douvere (2017) World Heritage marine sites: best practice guide.
Web portals with collections of resources on protected area management^b^
Equator Initiative (2017) Case study database, 37
EUROPARC Federation (2017a) Toolbox, 73
Global Transboundary Conservation Network (2017) Case studies, 14
National Biodiversity Strategies and Action Plans Forum (2015) Best practices, 124
Oppla (2017) Case study finder, 22
PANORAMA (2017) Solutions for a Healthy Planet: Protected Areas portal, 151
#NatureForAll (#NatureForAll) Success stories
Trainings and tutorials, including in-person courses and resources available online
Conservation Measures Partnership (2017)
The Nature Conservancy (2017). ConservationTraining
Warner College of Natural Resources (2012) Center for Protected Area Management and Training
National Conservation Training Center (2017) The National Conservation Training Center
Peer-reviewed journal articles including multiple case studies on ≥ 1 PA in an ecoregion or country^c^
Crabbe (2014), Capacity building and policy development in Belize marine protected areas, an example for Caribbean integrated coastal management
Havard et al. (2015), Stakeholder participation in decision-making processes for marine and coastal protected areas: Case studies of the south-western Gulf of California, Mexico
Stringer and Paavola (2013), Participation in environmental conservation and protected area management in Romania: A review of three case studies

^a^IUCN = International Union for the Conservation of Nature

^b^Numbers of case studies on PA management as of 8 November 2017, when known, are given

^c^Based on literature search in Web of Science using the following search keywords: protected area* and (“case studies” or examples). Papers published before 2013 or lacking descriptions of strategies to achieve socioecological sustainability of PAs are not included in the table

Based on theories drawn from learning sciences (Bloom 1956; Krathwohl 2002), psychology (Anderson 1996), and environmental conservation (Biggs et al. 2012), we propose that structuring a solution by breaking it into potentially replicable component parts (i.e., building blocks) will facilitate communication, learning, and adoption of one or more building blocks by other PA stakeholders. We further anticipate that providing PA stakeholders with a systematic approach to share structured solutions will inform, connect, and motivate PA stakeholders, and thus help overcome key challenges in PA management (IUCN 2015). Learning from peers about specific examples of successful management practices and governance arrangements provides an important means toward innovation and implementation of increasingly effective management strategies (Fish and Walton 2013).

**Fig. 1:**
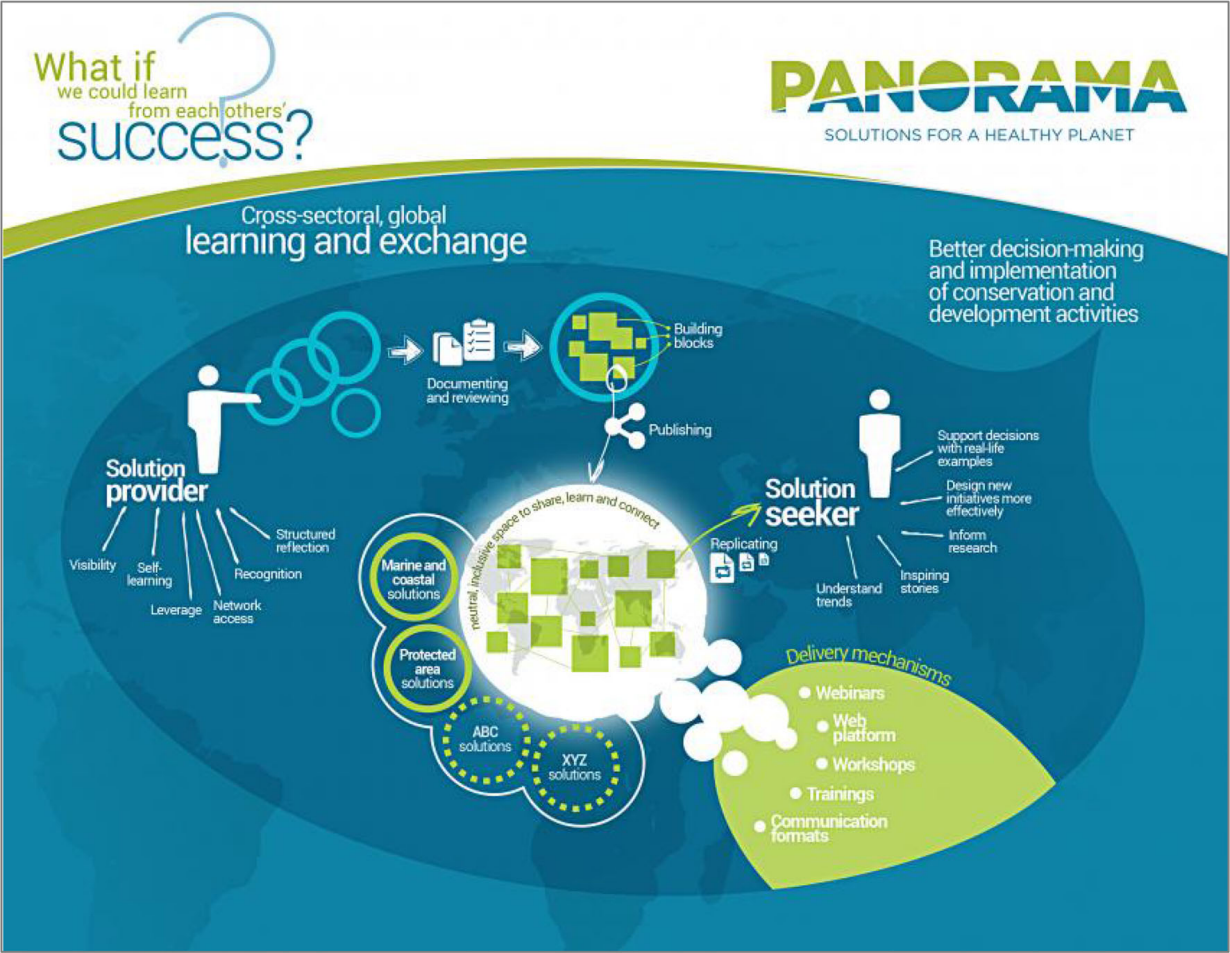
Schematic illustrating the PANORAMA—Solutions for a Healthy Planet initiative, which implements a solutioning approach via an online web portal, workshops, webinars, and trainings to support protected area management

Our general aim is to present a “solutioning” approach for sharing knowledge among conservation practitioners regarding examples of successful management in and around PAs. The term was borrowed from the fields of counseling (Webb 1999) and information technology (Malik 2013) to describe a process that engages peers and social discourse to seek answers to questions about behavior change and innovation. Although solutioning can be applied by a diverse range of actors, we focus here on the application of solutioning by PA stakeholders addressing issues within and beyond their protected area. We will begin by describing theories to support this approach followed by a description of the PANORAMA—*Solutions for a Healthy Planet* initiative (henceforth, PANORAMA; Fig. 1), which has pioneered and implemented this approach in the context of PA management. In particular, we illustrate the design of PANORAMA, including its online and offline community of users who engage through four phases that comprise solutioning. We also highlight solutions that have been published and have inspired others to enact parts of them in their own regions. In closing, we outline the future vision for PANORAMA and how this approach could inform other parallel efforts to support socioecological sustainability within and beyond PAs.

### Solutioning: Definition and theoretical foundations

**Fig. 2:**
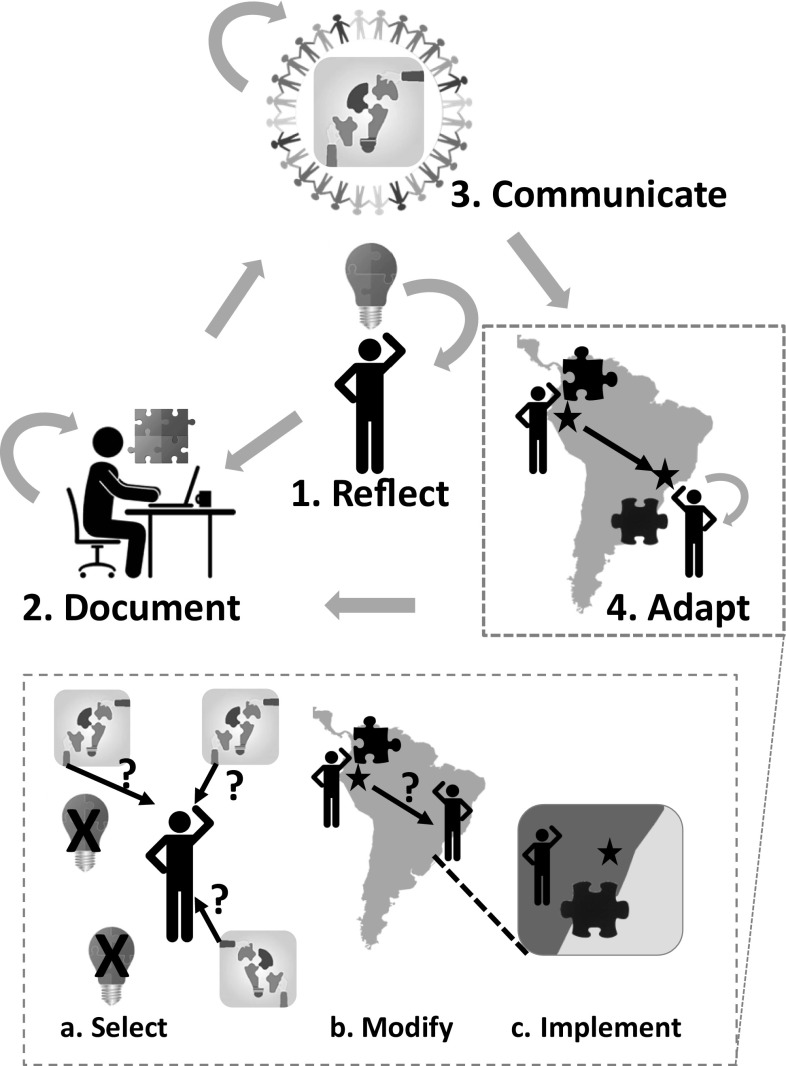
The solutioning process as applied to protected area (PA) management. One or more PA stakeholders carry out the four phases of solutioning as follows: (1) reflect on a particular solution that they have implemented to address a problem in one or more regions; (2) document the problem, solution, and publish including the component building blocks; (3) communicate the solution with peers via publications, webinars, and workshops; and (4) adapt building blocks of an original solution and implement them in other region(s) where they work The last phase involves three steps for a PA stakeholder: (*a*) select among existing solutions or individual building blocks that are relevant to the issues they are facing; (*b*) modify the selected building blocks as needed for proper implementation in the new context; and (*c*) implement the adapted building block, often requiring a participatory approach that involves the relevant stakeholders

We introduce solutioning as a four-phase process to address one or more challenges facing protected area stakeholders aiming to support objectives for nature and people (Fig. 2). Although we focus on the application of the approach by PA stakeholders, solutioning may be enacted by any conservation and sustainable development practitioner. This approach is supported by theories of knowledge transfer, peer learning, and social-ecological resilience. We briefly describe each of these main theoretical foundations.

The well-known Bloom’s Taxonomy of Learning (Bloom 1956) acknowledged that learning from dissimilar examples can be difficult. Extrapolation from a single example was identified as the highest form of comprehension, and synthesis of multiple examples to produce a new idea and apply to a novel situation was identified as one of the most difficult learning tasks of all. More recently, Krathwohl’s (2002) revision of the taxonomy proposed that knowledge about when and how to apply principles was the highest form of procedural knowledge. To help PA managers apply lessons from seemingly disparate case studies, it may therefore be useful to seek ways to simplify the learning task.

Psychologists have long embraced Bandura’s (1985) theory of social learning, which posits that humans learn best by replicating the behaviors, norms, and beliefs of valued “others.” However, as the behaviors become more complex, imitation grows more difficult. Anderson’s (1996) Adaptive Character of Thought Theory argues that humans learn new approaches to complex problems through the acquisition and interaction of procedural and declarative knowledge. Declarative knowledge is obtained in units called “chunks” that are simple enough to be processed, while procedural knowledge describes how things are connected together in one’s environment. By breaking management problems into potentially replicable component parts and providing clear guidance on how to carry out a solution in a stepwise fashion, solutioning provides a way to help PA managers recognize and process the chunks from which learning can be achieved.

Sharing knowledge among peers through communities of practice can be especially effective for learning, innovation, and implementation of novel management strategies (Reed et al. 2014). By breaking down and documenting a solution according to its component building blocks, tacit (i.e., undocumented) knowledge is converted to explicit (i.e., documented) knowledge (Nonaka and Takeuchi 1995) that can then be transferred between PA stakeholders. Within a conservation organization or natural resource management agency, the solutioning approach encourages knowledge creation through development of new solutions and building blocks along with knowledge and retention through the documentation and storage of these solutions and building blocks (Argote and Miron-Spektor 2011). Through the transfer and uptake of building blocks in a novel application domain or administration, practitioners consider a broader range of management approaches in different contexts. This process should therefore generate improved understanding and enhanced capabilities for adapting and improving the solutioning process within a particular community of practice.

Biggs et al. (2012) proposed seven principles (P1-7) for enhancing resilience of ecosystem services in the face of disturbances and dynamics of social-ecological systems (SES), and we argue that the solutioning approach is useful for realizing at least some of these principles across diverse ecosystems and multiple spatial and temporal scales. First, it encourages learning and experimentation (P5) through sharing lessons learned among PA stakeholders regarding the challenges and successes of implementing solutions (and their component building blocks) in diverse contexts. Below, we expand further on the theory of organizational learning and how this relates to the solutioning approach. Second, maintaining diversity and redundancy (P1) in SES can be achieved through ensuring heterogeneity of biological communities and management approaches. Although methods for maintaining biological diversity must be tailored to the particular areas where they are applied (Ferraro and Hanauer 2015), the fields of natural and social sciences offer general theories, and conservation organizations have prepared general guides (e.g., IUCN 2016) that can be used as a basis to formulate building blocks that are transferable. Lastly, when applied to protected area management, solutioning broadens participation (P6) by engaging networks of PA stakeholders in developing, implementing, documenting, and sharing their solutions and building blocks.

## Panorama initiative: Overview

The PANORAMA—Solutions for a Healthy Planet initiative began in 2014 (henceforth, PANORAMA; Appendix S1; Fig. 1). It addresses the need to understand and analyze what constitutes success in PA management through the four phases of solutioning (Fig. 2). PANORAMA serves a wide variety of institutions and individuals, and it serves as a mechanism for sharing solutions and their component building blocks along with lessons learned. PANORAMA is coordinated by German International Cooperation Agency (GIZ) and International Union for the Conservation of Nature (IUCN), and it is being implemented in partnership with United Nations Environment Programme, GRID-Arendal, and Rare (a non-governmental conservation organization). Although PANORAMA facilitates solutioning within and beyond the thematic realm of protected area related issues, the purpose of our study is to describe the solutioning process as it applies to solutions that include PAs.

### Web platform

PA stakeholders publish their solutions and view others’ solutions on the PANORAMA web platform, particularly the thematic portal on “Protected Areas.” The PANORAMA platform currently has 3 further thematic portals: “Ecosystem-based Adaptation,” “Marine and Coastal,” and “Agriculture and Biodiversity,” with further themes foreseen to be added in the future. Each theme is coordinated by an organization, sub-unit of an organization, or a consortium (e.g., IUCN Global Protected Areas Programme for the “Protected Areas” community). All thematic portals are part of a single database storing all solutions and building blocks. In addition to viewing solutions by entering through any of the thematic portals, users can view all solutions within that database on the “Explorer” page (PANORAMA 2018). The platform provides diverse means of search and filtering, e.g., by ecosystem or region. It is built on the web content management platform Drupal.

### Describing and refining a solution for publication

Solutions, as defined in the context of PANORAMA, are specific, applied examples of successful processes or approaches to protected area management and governance. They can represent entire projects or only aspects of a project, and they typically encompass several phases of activities. Recognizing the global scope of PANORAMA, solutions can be published in any of 3 languages (EN, FR, or ES). They are documented in a way that is understandable for audiences from diverse cultures, to enhance the opportunity for building blocks to be adapted for new contexts. Documenting a solution is the second step in the solutioning process, following the initial step of self-reflection and summarizing key factors that made their work successful (Fig. 2).

Before being published on the PANORAMA web platform, each solution is subjected to a review process, conducted by IUCN staff members and in some cases an external reviewer designated by IUCN (e.g., member of the IUCN World Commission on Protected Areas). The reviewer provides comments to support the solution provider in meeting the required quality standard, particularly with regard to general logic, clear description of the core idea, selection and description of building blocks, clarity of expression and grammar, and adherence to the format.

Any PA solution to be considered must meet the following criteria: (1) thematically relevant: solutions respond to challenges for sustainable development and human well-being and contribute to maintaining or improving biodiversity and ecosystem services in one or more protected areas; (2) impactful: solutions are effective and their implementation shows strong potential for improvements in ecological, social, and economic conditions; and 3) replicable and/or scalable: building blocks of the solution have the potential for adaptation, replication or upscaling in other geographic, social or sectorial contexts.

Solutions on the PANORAMA web platform are documented in a standard format as either a “full solution” or as a “snapshot solution” (Appendix S2). Here we focus on the full-solution template, as the snapshot solution template is a subset. The full-solution template starts with general information including the solution title, names of the contributors, world region, specific location(s) where the solution was implemented, summary, positive social, ecological and/or economic impacts, images illustrating the solution, and links to related resources.

The remaining sections of the template are required for full but not snapshot solutions, and these sections include challenges addressed, list of beneficiaries, ecosystem types, themes covered, scale of implementation, organizations involved, and a personalized story to highlight certain aspects of the solution (Table 2; Appendix S2). Entries for the relevant world regions (*n* = 16), ecosystems (*n* = 40), and themes (*n* = 67) represent the primary dimensions of the solution and provide the basis for a guided filter mechanism on the web platform. In addition to the introductory sections, up to 6 building blocks need to be described by completing the following sections for each building block: title, summary description, enabling factors, lessons learned, classification of building block, scale and phase of implementation in the context of the overall solution, images, and links to relevant resources. The final section of the template is a description of how these building blocks interact to produce the solution as a whole. Solutions are therefore formatted to achieve the overarching goals of the PANORAMA initiative, as they summarize impacts on socioecological sustainability within a particular context including individuals and organizations involved along with the lessons learned from implementing the building blocks.

**Table 2:** Hypothetical selections of solution dimensions by a solution seeker and corresponding examples of protected area solutions published on the PANORAMA—Solutions for a Healthy Planet web platform

Hypothetical selections of solution dimensions^a^				Example solution matching each hypothetical set of dimensions	
Region	Ecosystem	Theme	Type	Title	Citation
Southeast Asia	Tropical deciduous forest	Outreach	Full	Integration of local knowledge in park management	Dobbelsteijn (2017)
East and South Africa	Mangrove	Indigenous people	Full	Improving relationships between local communities and Saadani National Park management	Downie (2017)
East and South Africa	Desert ecosystems	Protected area governance	Full	Making protected area concessions work for communities	Snyman (2017)
West and Central Africa	Temperate evergreen forest	Sustainable livelihoods	Full	Conflict resolution strategy for Kahuzi-Biega National Park	Kujirakwinja (2017)
Europe	Temperate deciduous forest	Connectivity/transboundary conservation	Full	Promoting transboundary co-existence of large carnivores	Mattsson and Vacik (2018)

^a^The full range of selections for each dimension are given in Appendix S2

### Communicating solutions and building blocks

In addition to visiting the PANORAMA web portal, PA stakeholders communicate about published solutions and building blocks through several pathways, including webinars, workshops, publications, newsletters, and social media. These modes of communication are designed to help practitioners share their stories in a consistent way, get recognized for successful work by other PA stakeholders, learn how others have tackled problems across the globe, and reflect and consider implementing new management approaches in their own context (Fig. 1). For example, the PANORAMA webinar series focuses on particular themes, seeking to provide a useful means to promote existing solutions, stimulate discussion about uptake of building blocks, and inspire PA stakeholders to reflect and submit new solutions of their own. One of the webinars was supported by an accompanying IUCN publication on that theme (i.e., transboundary conservation; Rodrigues and Fischborn 2016). The webinar recordings and individual solutions are further highlighted via newsletters and social media.

As another form of live interaction through PANORAMA, IUCN and partners have hosted solutioning workshops to discuss existing PA solutions and building blocks to promote learning and to initiate adaptation and uptake of building blocks (Appendix S3). In-person workshops offer benefits for both solution providers and for solution seekers. For solution providers, the workshop provides a vehicle to potentially transfer and adapt their solutions to new application domains. For solution seekers, the solutions and building blocks discussed during the workshop provide inspiration for finding ways to address their own challenges. Beyond the exchange of knowledge, solutioning workshops also have clear networking benefits resulting from the diverse expertise and knowledge of the participants.

PANORAMA solutions and building blocks are integrated into training modules. The use of solutions in a training event supports the learning process, as they serve as examples from practice that complement the theory of training content. The specific theme of the training provides the audience with an overall framework, while the case studies within that theme address context and detailed consideration of local needs, and/or can enrich the discussions by illustrating relevant lessons from similar or other geographic contexts.

### Transfer and uptake of building blocks

As predicted by learning theories (Anderson 1996; Krathwohl 2002), the last and most time-intensive phase in the solutioning process involves adapting one or more building blocks from existing solutions for implementation in another situation (Fig. 2). A PA stakeholder reflects on existing solutions or building blocks to determine whether these can be adapted for implementation in their own context, and following consultation and deliberation, applies these ideas in practice. By learning about solutions and building blocks through the PANORAMA web platform, the webinars and the discussion at the workshops, PA stakeholders gain insights into how individual building blocks have been applied in the original context. Through further reflection, the building blocks can be adapted to a new local context, taking advantage of the knowledge shared from colleagues in the PANORAMA community of practice. Implementing the adapted building blocks has potential positive outcomes for biodiversity and human livelihoods, and these outcomes should be monitored to support learning and future improvements.

## Panorama initiative in action

**Fig. 3:**
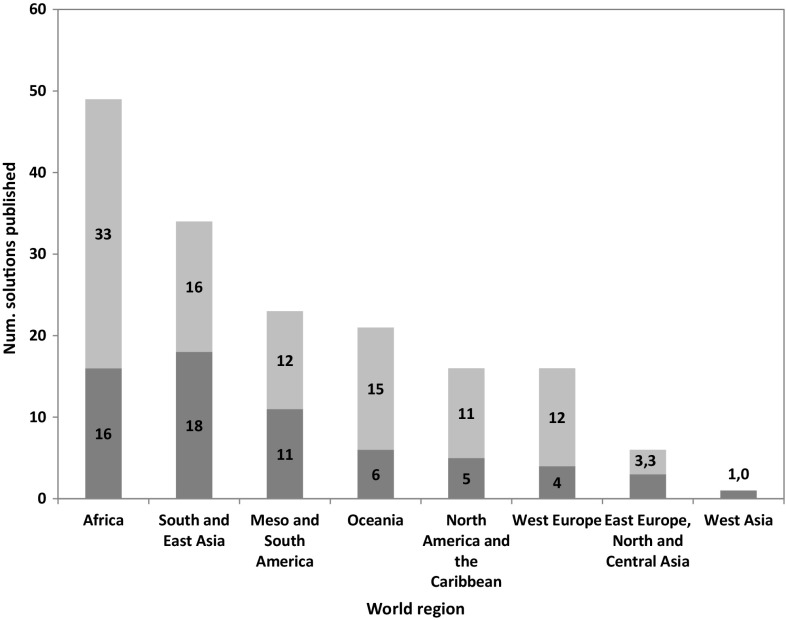
Geographic distribution of solutions published on the PANORAMA—Solutions for a Healthy Planet protected areas web portal as of March 2017. Dark-shaded bars represent full solutions, and gray-shaded bars represent snapshot solutions

Although PANORAMA has only recently been developed, there has been a large degree of participation in the initiative. As of February 2018, 369 solutions were published on the web platform across the currently 4 thematic portals, including 270 full solutions (i.e., solution summary plus at least two building blocks) and 99 Snapshot Solutions (i.e., solution summary) describing conservation and natural resource management solutions distributed across all continents except Antarctica. Of these, the “Protected Areas” portal feature 200 solutions, 118 of which are full solutions, and 82 are snapshot solutions (Fig. 3). On average, 1 full solution and 2 snapshot solutions have been submitted each month for publishing on the “Protected Areas” portal. In addition to the publishing activities, during 2016 and 2017 there were 9 bi- to tri-monthly “protected area solutions” webinars averaging 115 registrants and 44 attendees, who then receive recordings of the presentations (Table 3). On average, 71 people viewed the recording of each session.

**Table 3:** Overview of protected areas (PAs) webinar series organized by PANORAMA—Solutions for a Healthy Planet

Topic	Date	Registrants who received the webinar recording	Attendees	Turnout ratio (%)	Number of users who viewed webinar recording (as of 06 November 2017)
African PA solutions to climate change	Jan 2016	72	34	47	56
PA tourism (session 1)	Mar 2016	77	55	71	103
PA tourism (session 2)	Apr 2016	88	39	44	59
Island solutions	Aug 2016	126	41	33	17
Engaging young people in PAs	June 2016	103	31	30	121
Transboundary PA solutions	Dec 2016	141	43	30	102
Gender mainstreaming solutions for PAs	Mar 2017	195	68	35	166
Scaling up community-led MPA management	June 2017	253	92	36	80
Solutions on lessons learned in the management of Amazon Protected Areas (Soluciones sobre lecciones aprendidas en la gestión de Áreas Protegidas Amazónicas)	Oct 2017	220	47	21	40

Several solutioning workshops have been conducted. The largest of these was a series of workshops entitled “Blue Solutions Regional Fora” that spanned several days and brought together over 100 practitioners in marine and coastal conservation. The focus was on local to regional-scale marine conservation efforts across a certain region, and the workshops included sessions for knowledge sharing on solutions relating to management of marine protected areas and other issues. Other workshops have been smaller-scale. For example, a half-day workshop with staff from IUCN, GIZ, and KfW (Kreditanstalt für Wiederaufbau) banking group focused on identifying (1) building blocks from protected area-related projects around equitable governance being implemented by local actors; (2) lessons learned from existing applications of the solutioning approach; and (3) ways of improving the solutioning approach in light of these lessons learned.

Although more challenging to document, multiple instances of building block adaptation and uptake have already occurred during the first years of the initiative. To illustrate the process, we describe two examples where one or more building blocks of a solution were adapted and implemented in another context. In the first example, multiple building blocks were adapted to several locations in Laos. Appendix S4 describes a building block implemented near the west coast of southern Africa that motivated thinking about potential building blocks to implement in another country along to the opposite coast of the continent. The second example illustrates how solutions can be transferred between countries.

### Case study: Building block adaptation between PAs of Laos

The management of Hin Nam No National Protected Area (HNPA), located along the Lao-Vietnamese border, had been facing many challenges, including lack of human and fiscal resources along with insufficient engagement of stakeholders in the surrounding communities regarding day-to-day management and decision-making (de Koning et al. 2016). One of the goals of protected area managers is to maintain biodiversity in the region, which requires managing hunting and poaching activities. Given that these activities occur within the local villages embedded within the protected area, effective management is only possible with strong participation and cooperation of stakeholders in the local communities. To address this need, staff of the German International Cooperation Agency (GIZ) worked with park authorities and local community members to enact the solutioning process (Fig. 2). Each step of the process was followed and in step 2 several building blocks were documented to comprise the solution: participatory mapping and zoning of the area, villager ranger training to enforce harvest and poaching policies, a monitoring system to track data related to poaching activities, and systematic wildlife observations (HNPA 2010; de Koning et al. 2016; Dobbelsteijn 2017). In step 3, building blocks within the HNPA solution were then shared with nine other PAs in Laos. To date three of these PAs have adapted these building blocks (step 4) to approve 21 village co-management agreements (Phommasane 2017), illustrating another case of building block adaptation between regions.

## Discussion and conclusions

We have presented solutioning as a process of reflection, documentation, communication, followed by adaptation and uptake of successful management in and around protected areas. The process is supported by theories from psychology, education, ecology, and conservation biology, and has a general aim to support the achievement of objectives of protected area authorities at a local scale and socioecological sustainability at broader scales. Recognizing the diverse objectives and contexts among regions, the process is designed to document tailored solutions and building blocks that are then adapted to other areas facing similar issues and challenges. The 2 examples facilitated by the solutioning methodology support the idea that, through the deconstruction of a solution into its adaptable components, solutioning can facilitate adaptation and delivery of existing successful approaches leading to improved ecological and social conditions.

### Successes

The PANORAMA Solutions for a Healthy Planet initiative has pioneered development of the solutioning process in the context of PA management, and has put it into practice through an interactive community and protected areas solutions web portal hosting over 200 PA solutions that have been implemented on every continent except Antarctica. This initiative is expected to persist and expand well into the future, with a general aim of expanding its network of influence to an increasingly diverse suite of site managers, NGO staffs, researchers and government agencies. PANORAMA partner organizations (e.g., Global Environment Facility and Germany’s Federal Ministry for the Environment, Nature Conservation, Building and Nuclear Safety) have committed financial support and encouraged their members to participate in the initiative (e.g., contributing solutions, joining webinars, and hosting workshops) in exchange for dedicated portals of solutions (on the PANORAMA website) related to the respective missions of the organizations. With this diverse support, the PANORAMA initiative is expected to expand and persist within the mandates of existing and future partner institutions (Appendix S5). In return for the external support, PANORAMA supports participating organizations and individuals in sharing knowledge on how to achieve goals for conservation and sustainability.

Looking more specifically beyond PANORAMA, other initiatives use standardized templates to collect and publish on-line case study descriptions of successful management in the fields of nature conservation and socioecological sustainability (Table 1). Our review finds that PANORAMA is the only such initiative that uses the solutioning process to develop a large portfolio of case studies on protected area management and governance around the globe. The solutions collectively describe very diverse approaches operating at multiple levels of implementation (from individual PAs to continental-scale efforts), which have been led by a wide variety of actors and institutions. PANORAMA is also the only one of these initiatives that uses a modular case study format, offering building blocks within solutions. This presents a challenge and an opportunity for PANORAMA to collaborate with these related web platforms and to share case studies despite the diverse templates among platforms.

Solutions, while context-specific, are seen as toolboxes that demonstrate successful application of a suite of building blocks and can be adapted across geographies and themes. The solutions within PANORAMA address diverse issues relating to biodiversity conservation in the context of sustainable development and human well-being. Solutions document building blocks that can be used to address Sustainable Development Goals (International Council for Science and International Social Science Council 2015), and such documentation can serve a basis for parallel certification processes (Boiral and Gendron 2011; Jaung et al. 2016) that acknowledge the progress that is being made on the ground by PA staff and stakeholders.

### Challenges for the future

In reviewing PANORAMA, we identified potential areas for improvement. One concern is the lack of quality indicators in the database of solutions. Currently, solutions are reviewed by a small group of IUCN staff members, and since August 2017, some solutions receive an external review by experts from the IUCN World Commission of Protected Areas’ membership. Having external review for every published solution would improve the quality of the database. Although registered users may post comments to individual solutions, there is currently no means to provide anonymous feedback (e.g., by clicking a “like” button). Displaying the level of external review and allowing for anonymous feedback on the solutions will provide indicators of quality. Another concern for the future is the webinar series, which is one of the main mechanisms (other than in-person workshops) to maintain live interaction with the user community. Although there were 6 webinars in 2016, there were only 3 in 2017 and so far none in 2018 (as of February; IUCN 2018a), which may be too infrequent to maintain or increase the set of participants. A third area for improvement is the linkage between the database of solutions and other elements of the initiative. Of the 9 webinars there have been only 2 that have had associated publications summarizing the relevant solutions (IUCN 2018b), which are an effective means of linking the webinars and database of solutions. The newsletters along with the list of webinars and associate publications are hosted on a separate IUCN website (IUCN 2018b), and there is no link to this website from the solutions explorer (PANORAMA 2018).

Beyond issues related to the solution-review process and webinars, mechanisms for stakeholder engagement should also be made clear for the PANORAMA initiative to achieve its ambitions of ensuring sustainable and equitable management in and around PAs. The solutioning approach offers ways to enhance stakeholder engagement, but stakeholder participation in PA solution-building and solution-transfer is not made explicit in the solutioning process. It is all too common in PA management for stakeholder outreach to be insufficiently broad, or for stakeholders to be engaged too late in the process (Agardy et al. 2011). The solutioning process is likely to work best when participation strategies are built into the framework from the start, and extra efforts are made to broaden participation beyond the most obvious stakeholders (Sayce et al. 2013). Further work is needed to identify optimal mechanisms for stakeholder engagement in the solutioning process under particular management contexts.

### Research opportunities

Nonetheless, the broad coverage and standardized template for solutions being published on PANORAMA offers research opportunities. In particular, researchers can use the on-line solutions and building blocks to examine hypotheses about whether the PANORAMA initiative is improving efficiency and equitability of PA management. Such investigations would be possible through increasing sophistication of tools for quantifying information flows via the internet (Lei et al. 2015). Two general kinds of investigations could be conducted. First, researchers can address questions about what determines knowledge transfer via the PANORAMA portal using web analytics to monitor visits to particular solutions and building blocks (Soriano-Redondo et al. 2017). Certain attributes (e.g., location of implementation, themes addressed) may attract more viewers to read about particular solutions or building blocks, which could guide future research on the design of supportive solutions. Second, researchers can examine spatial and temporal patterns in the objectives and actions being taken by protected areas and associated stakeholders by using formal content analysis (Bhatia et al. 2013; Jiménez et al. 2015). Such an analysis could reveal insights into contrasting goals and strategies among regions and how these relate to targets under the Sustainable Development Goals (International Council for Science and International Social Science Council 2015), Aichi Biodiversity Targets of the Convention on Biological Diversity (CBD 2013), and other relevant global to national-scale policy commitments. Such an endeavor could complement parallel efforts examining protected area management effectiveness using standardized databases and approaches (Brooks et al. 2015; Coad et al. 2015).

Although social science theory suggests the solutioning approach creates a better process for finding solutions and engages stakeholders in a way that should lead to more effective solutions (Jamal and Stronza 2009; Sayce et al. 2013), this assumption has not been tested. Such evaluation is outside the scope of this paper. However, we suggest that the framework for management effectiveness developed by the IUCN World Commission for Protected Areas (Hockings et al. 2006) would provide an appropriate lens through which to assess contributions of the solutioning approach for addressing challenges in protected area management. For example, one might draw a random sample of protected area cases from the PANORAMA database, pair each one with a similar protected area where the solutioning approach had not been used, and compare outcomes between each pair for the six assessment categories identified by Hockings et al. (2006): context, planning, inputs, management processes, outputs and outcomes.

### Learning, adaptive management, and transformation

Our analysis suggests that the solutioning approach, operationalized through PANORAMA, can be useful to government agencies responsible for protected area management. Assessing, understanding and summarizing “what works” in protected area management and governance will promote learning while providing valuable information to inform future policies and reporting on progress to international targets, such as the CBD’s Aichi Target 11. An example of adapting building blocks within a governmental conservation agency has occurred in the upper midwestern US, where a network of 22 national wildlife refuges managed by the U.S. Fish and Wildlife Service has worked with scientists in the U.S. Geological Survey to develop and implement a joint adaptive management program that has enhanced their effectiveness at managing invasive plant species to maintain biodiverse prairie habitat across an ecoregion (Moore et al. 2013).

Learning and adaptive management can promote effective conservation planning and natural resource management in diverse contexts (Grantham et al. 2010; Williams and Brown 2012). Non-governmental conservation organizations (NGOs) such as Rare have developed approaches to achieve transformational behavior change by borrowing from psychology, marketing theory and other social sciences disciplines in identifying so-called “bright spots,” i.e., locally led solutions, and repeat them in communities around the world (Rare 2015). These approaches are expected to bring about voluntary behavior changes inspired by specific, locally owned solutions. This example illustrates that conservation agencies and organizations can move beyond repeating traditional conservation and natural resource management practices that have been locally established.

Ideally, the collaborations and stakeholder engagement fostered by the solutioning process would not only benefit individual protected areas and associated stakeholders, but lead to improvements in socio-ecological sustainability at broader scales. The initiative’s creators envision that it can be a catalyst for change and transformative governance toward socioecological sustainability (Chaffin et al. 2016). The text published in the standardized template for solutions and building blocks on the web platform along with on-ground verification procedures can be used to evaluate this hypothesis. If so, solutioning could therefore create positive impacts for science and society at the local level and broader political levels, by encouraging and motivating individuals, communities and institutions to take positive and documentable action for socioecological sustainability.

## Electronic supplementary material

Below is the link to the electronic supplementary material.

Supplementary material 1 (PDF 157 kb)
