# Supplementary material for: Introducing and evaluating a knowledge transfer approach to support problem solving in and around protected areas
Source: Ambio. 2018 Mar 30;48(1):13–24. doi: 10.1007/s13280-018-1048-5 (PMC6297101; doi:10.1007/s13280-018-1048-5)
Supplement: Supplementary file 1 — Supplementary material 1 (PDF 157 kb) [file 13280_2018_1048_MOESM1_ESM.pdf]

Ambio

Electronic Supplementary Material

This supplementary material has not been peer reviewed.

Title: **Introducing and evaluating a knowledge transfer approach to support problem solving in and around protected areas**

Authors: Brady J. Mattsson, Marie Fischborn, Mark Brunson, Harald Vacik

Appendix S1. History of the PANORAMA initiative.

The PANORAMA – Solutions for a Healthy Planet initiative builds on conceptual work led by the International Union for the Conservation of Nature (IUCN) World Commission on Protected Areas and partners, profiling protected areas as "natural solutions" to global challenges such as climate change (IUCN 2015). As an investment laying foundations for PANORAMA, the Blue Solutions project (https://bluesolutions.info) pioneered the solutioning approach, developed in partnership between the German International Cooperation Agency (GIZ), GRID-Arendal, IUCN and United Nations Environment Programme (UNEP), with a focus on conservation and sustainable use of marine and coastal resources (not limited to protected areas). This partnership led to development of a case study template and methodology for documenting and sharing solutions, which was adopted for the newly conceived PANORAMA initiative that was to initially focus on protected-area (PA) management and governance.

With funding provided by the Global Environment Facility, IUCN's Global Protected Areas Programme in collaboration with the United Nations Development Programme led a project with the aim of using the IUCN World Parks Congress 2014 as a 'strategic platform for development & learning' and supporting existing and new PA learning networks. PANORAMA was an integral part of that project. PANORAMA as an initiative was formally launched at the Congress, with an initial focus on protected area solutions to complement the contemporary Blue Solutions. The beta PANORAMA web platform was presented during the Congress, with an initial set of around 40 "inspiring protected area solutions". IUCN, at the Congress, highlighted the opportunity for PA professionals to document and share their solutions to foster an emerging global community of practice. In addition to the presentation, IUCN staff approached presenters at the Congress to solicit their solutions. Many of these presenters agreed and submitted their solutions for inclusion using the provided template. Following the Congress, PANORAMA was carried on as an ongoing effort. Solutions were continuously being documented and published on the PANORAMA portal. These solutions were initiated by proactive submissions from solution providers that had heard about

PANORAMA or by IUCN staff contacting potential providers based on peer recommendations and information published elsewhere.

The process for publishing a solution typically followed four steps: 1) provider filled the template and submitted this as draft; 2) IUCN staff reviewed the draft and provided comments; 3) solution provider revised the draft according to the comments and resubmitted it; and 4) solution was published once both sides had agreed on a final version. During the first step, IUCN staff would sometimes assist the solution provider to clarify any questions they had when initially filling the template. In some cases IUCN staff or other PA professionals used available documents (e.g., final reports from projects) to prepare an initial draft solution, which was then followed by step 3 above. IUCN staff worked in close coordination with the provider to ensure that the text and images were suitable for the solution template, and as such Steps 2 and 3 were often iterative to accommodate multiple rounds of edits by the provider if needed. The solution description was always original writing, even though it was sometimes based on existing websites or publications.

Following feedback from early users of the beta PANORAMA web platform and users of an initial Blue Solutions platform, a much improved platform was developed and then released and presented at the IUCN World Conservation Congress in 2016, initially hosting the “protected area solution” case studies assembled by IUCN thus far, as well as “marine and coastal solution” case studies assembled through the Blue Solutions project. With this new iteration, PANORAMA was framed as a broader initiative, not only as a web platform but also an effort to engage conservation practitioners and develop partnerships through workshops, trainings, webinars and other means. Importantly, the thematic focus of PANORAMA was expanded beyond “protected areas”, recognizing that the “solutioning approach” is applicable across subjects, and inviting in new partners to apply the approach to new themes and join the partnership as coordinators for a particular theme, which are reflected as “portals” on the web platform. Building on the “Protected Areas Solutions” and the “Marine and Coastal Solutions” themes, a 3rd thematic community on “Ecosystem-based Adaptation Solutions” was launched at the Conference of the Parties to the Convention on Biological Diversity in Mexico, December 2016. Further thematic communities are expected to be added in 2017 and beyond. Today, PANORAMA is a partnership initiative to document and promote examples of inspiring,

replicable solutions across a range of conservation and development topics, enabling cross-sectoral, cross-thematic learning and inspiration. Following its evolution and the pioneering role of GIZ and IUCN in PANORAMA's development, these 2 organizations act as Founding Partners with a longer-term institutional commitment, coordinating and leading the partnership and its further development, ensuring its sustainability including through establishment of new collaborations, integration into programmatic priorities, staff and financial support, and overseeing the web platform.

Appendix S2. Template for full solution on PANORAMA web portal.

Character limits when relevant are given in parentheses, and an asterisk (*) indicates a required field.

1. Overview

Language [English / French / Spanish]

Portal * [Protected areas / Marine and coastal / Ecosystem-based adaptation]

Title *

Location on the map * Please name the place, country or region of implementation of your solution.

In case your solution is implemented in more than one location, please enter these locations here (250)

Summary * (500) Please write a short description that explains what the solution is and how it is being applied.

Impacts * (1400) Please describe the demonstrated positive impacts of your solution including environmental, social and economic impacts.

Related resources Add picture, video, document, link(s)

2. Challenges and beneficiaries

Challenges *(1000) Please describe the main challenges your solution addresses and, if possible, differentiate between environmental, social and economic challenges.

Beneficiaries * (250) Please name the beneficiaries of your solution.

3. Categories

Region * Check all that apply: Africa; East and South Africa; North Africa; West and Central Africa; America; Caribbean; Central America; North America; South America; Asia; East Asia; South Asia; Southeast Asia; West Asia Middle East; Europe; Oceania

Ecosystem * Check all that apply: Agro-ecosystem; Agroforestry; Cropland; Orchard; Rangeland / Pasture; Urban Ecosystem; Green roofs / Green walls; Green spaces (parks, gardens, urban forests); Urban wetlands; Marine and coastal ecosystems; Mangrove; Open sea; Rocky reef / Rocky shore; Lagoon; Estuary; Coastal forest; Coral reef; Deep sea; Salt marsh; Seagrass; Seamount / Ocean ridge; Beach; Forest ecosystems; Temperate deciduous forest; Temperate evergreen forest; Tropical deciduous forest; Taiga; Tropical evergreen forest; Freshwater ecosystems; Wetland (swamp, marsh, peatland); Pool, lake, pond; River, stream; Grassland ecosystems; Temperate grassland, savanna, shrubland; Tropical grassland, savanna, shrubland; Tundra, montane grassland; Desert ecosystems; Coastal desert; Cold desert; Hot desert; Other

Theme * Check all that apply: Biodiversity; Species and extinction; NBSAP; Biodiversity mainstreaming; Access and benefit sharing; Genetic diversity; Habitat fragmentation and degradation; Invasive alien species; Climate change; Disaster risk reduction; Adaptation; Mitigation; Communication; Environmental education; Outreach; Ecosystem conservation; Connectivity / transboundary conservation; Ecosystem services; Erosion prevention; Restoration; Governance; Policy and legislation; Political collaboration; Protected area governance; Human development; Sustainable livelihoods; Peace and human security; Health and human wellbeing; Food security; Cities and infrastructure; Infrastructure maintenance; Infrastructure maintenance; Local communities; Traditional knowledge; Indigenous people; Management planning; Watershed management; Land management; Urban planning; Spatial planning; Coastal and marine spatial management; Fire management; Flood management; Protected area management planning; Sustainable resource use; Sustainable tourism; Water provision and management; Agriculture; Responsible mining; Forest Management; Sustainable fisheries and aquaculture; Transport; Maritime transport; Terrestrial transport; Waste and resource efficiency; Marine litter; Pollution; Wastewater treatment; Waste management; Renewable energies;

Uncategorized; Sustainable financing; Standards / certification; Gender mainstreaming; Islands; Science and research; Poaching and environmental crime

Scale of implementation * Check all that apply: Local; Subnational; National; Multi-national; Global; Other

Contribution to international targets   Check all that apply:

Aichi targets

Target 1: Awareness of biodiversity increased

Target 2: Biodiversity values integrated

Target 3: Incentives reformed

Target 4: Sustainable production and consumption

Target 5: Habitat loss halved or reduced

Target 6: Sustainable management of aquatic living resources

Target 7: Sustainable agriculture, aquaculture and forestry

Target 8: Pollution reduced

Target 9: Invasive alien species prevented and controlled

Target 10: Ecosystems vulnerable to climate change

Target 11: Protected areas

Target 12: Reducing risk of extinction

Target 13: Safeguarding genetic diversity

Target 14: Ecosystem services

Target 15: Ecosystem restoration and resilience

Target 16: Access to and sharing benefits from genetic resources

Target 17: Biodiversity strategies and action plans

Target 18: Traditional knowledge

Target 19: Sharing information and knowledge

Target 20: Mobilizing resources from all sources

Sustainable development goals Check all that apply:

SDG 1 – No poverty

SDG 2 – Zero hunger

SDG 3 – Good health and well-being

SDG 4 – Quality education

SDG 5 – Gender equality

SDG 6 – Clean water and sanitation

SDG 7 – Affordable and clean energy

SDG 8 – Decent work and economic growth

SDG 9 – Industry, innovation and infrastructure

SDG 10 – Reduced inequalities

SDG 11 – Sustainable cities and communities

SDG 12 – Responsible consumption and production

SDG 13 – Climate action

SDG 14 – Life below water

SDG 15 – Life on land

SDG 16 – Peace, justice and strong institutions

SDG 17 – Partnerships for the goals

Other targets

4. An inspiring story

Please illustrate your solution by providing an inspiring, personal story of the solution in action. (2500)

Upload an image (e.g. of the story-teller) to illustrate the story.

5. People and organisations involved

Contributors

Contributor's full name * (75)

Contributor's organization (100)

Contributor's email

Add another contributor

Organizations Involved

Organization name * (100)

Organisation's website URL

Organisation logo

Add another organization

6. What are the solution's Building Blocks

Building blocks (BBs) are the core components of your solution such as instruments, tools, approaches, partnerships or processes that you believe could be applied in other contexts. They may be adapted and recombined with building blocks from other solutions to solve new challenges. A minimum of two building blocks is required, and a maximum of six building blocks can be uploaded.

Title *

Description *(1250) Please explain the purpose of this building block and how it works.

Enabling factors * (500) Please name conditions that are important to enabling the success of this building block.

Lessons learned *(1000) Please list key lessons learned during the process of implementing this building block. If relevant, please also mention aspects that have not worked and any specific advice to help anyone wishing to replicate your building block avoid being confronted with the same challenges.

Categories * Check all that apply: Alternative livelihoods; Capacity development; Co-management building; Collection of data and information; Communication, outreach and awareness building; Enforcement; Finance scheme; Law and regulations; Monitoring / evaluation / overseeing implementation; Partnership; Policy advocacy; Review; Stakeholder dialogue; Strategy and plan; Technical method, technique and tool; Other

Scale of implementation * Check all that apply: Local; Subnational; National; Multi-national; Global

Phase of solution Check all that apply: Planning phase; Inception phase; Implementation; Monitoring; Documentation and dissemination of results; Review phase; Entirety

Add picture

Add Links

Add new Building Block

7. How do the building blocks interact?

Description (1250) Please briefly describe how the building blocks interact with each other in your solution to address the challenges. Step-by-step instructions or a pictorial chart may be helpful for greater clarity.

Appendix S3. PANORMA solutioning workshop formats.

Solutioning workshops have been useful in two general formats: one is where only staff from a single organization participate, and the other is where several actors representing each of multiple regional organizations participate.

- The single-organization workshops enable evaluation of existing knowledge and its sharing within the staff of the organization, where individuals reflect on their experience before discussing solutions and building blocks that can be transferred within the organization and applied on the ground.
- The multi-organization workshops allow actors to connect not only around shared issues and geographic contexts, but also across sectors and geographies, which supports the upscaling of solutions e.g. across a broader region.

It is also possible to conduct solutioning workshops at a site level, discussing solutions with stakeholder representatives, including PA authorities, representatives of non-government organizations working in the area, and local business owners adjacent to a protected area.

Appendix S4. Building block adaptation between coasts of Africa.

This example illustrates the application of each step of the solutioning process (Fig. 1 in main text). The process began in step 1 with a privately owned wilderness safari and the Torra Conservancy, which are stakeholders involved with a community-run protected area in Damaraland near the northern coast of Namibia, reflecting on a solution they had implemented. The region had been facing biodiversity losses caused by overhunting of local wildlife for bushmeat and for protection of livestock, and the situation was exacerbated by extreme poverty and harsh climatic conditions. In response to these challenges, a private wildlife tourism company formed a joint venture with the Torra Conservancy to support socioecological sustainability for Damaraland. This joint venture led to a boost in the local economy through creation of jobs associated with wildlife tourism and improvements in biodiversity following recovery of impacted wildlife species, including Hartman's mountain zebra (*Equus zebra hartmannae*), African bush elephant (*Loxodonta africana*), and black rhino (*Diceros bicornis*). After this reflection, the stakeholders documented and communicated the solution in steps 2 and 3 of the solutioning process (Snyman 2017)

Meanwhile along the opposite coast of Africa, communities in the Saadani region along the northern shore of Tanzania were facing challenges following establishment of Saadani National Park in 2005. In particular, the local communities expressed concerns that they had lost access and use rights to the land and were therefore unable to benefit from the creation of the protected area (Downie 2015, Kesho Trust 2017). Recognizing the need and potential for using solutioning to further progress in improvement of relations between the PA and surrounding stakeholders in the Saadani region, a series of two PANORAMA workshops were held that included staff from IUCN and Kesho Trust (non-government organization [NGO] supporting community-based conservation), international sustainable tourism experts network (Linking Tourism & Conservation), authorities from Saadani National Park, staff from a local NGO (Saving Africa's Nature), and representatives of six local villages (Downie 2016). During these workshops the participants were introduced to published PANORAMA solutions including the one from Damaraland (Kujirakwinja 2017, Snyman 2017), and they discussed how the published building blocks might be adapted and applied (step 4) to address the identified challenges

within the Saadani region. The community participants also identified building blocks on their own, facilitated by Kesho Trust, which has led to gradual improvements in the relationship with the park administration over the last few years. This solution was later published on the PANORAMA platform (Downie 2017).

Here, we highlight building blocks from the Damaraland solution that provided inspiration in the Saadani context. First a plan for a joint venture was initiated between local communities and a private tourism company in the Saadani region, which was directly inspired by the Damaraland solution, in addition to input from all groups involved in the workshops. Second, they identified a suitable site to create a wildlife tourism camp in Saadani, which was also inspired from the Damaraland solution. Although the joint venture and associated tourism camp have yet to be implemented, this example from Africa illustrates how a building block for a solution in one region (here, Damaraland) can inspire the design of a solution in a distant region (here, Saadani National Park) facing similar challenges and finally leading to an adapted building block being implemented.

Appendix S5. Future prospects for the PANORAMA initiative.

Partnering with additional institutions is critical for maintaining and expanding the PANORAMA initiative, recognizing the increasing costs of maintaining updated website technology along with meeting changing needs and interests of funders and protected area professionals. New thematic communities, reflected through portals on the web platform, will continuously be added to PANORAMA based on interest of partners and their commitment to coordinate the solicitation and publication of solutions within these portals. At the discretion of the PANORAMA steering group, partner organizations will additionally be asked to provide financial and in-kind contributions.

The steering group also defines priorities and criteria for new themes and partners – including a shared „sense of responsibility“ for sustaining PANORAMA. The goals of additional thematic communities are to broaden the thematic scope and the partnership base of PANORAMA to achieve a more diverse set of solutions, facilitating inter-sectorial learning, and expand the inclusiveness and longevity of the PANORAMA initiative. For example a new portal on sustainable agriculture is scheduled to be launched mid-November 2017, coordinated by Rare and GIZ, and further thematic communities are under discussion. As another example, within the protected area portal, there are 17 solutions that are relevant to transboundary natural resource management (Rodrigues and Fischborn 2016), and a partner organization could offer to coordinate a new portal on this topic to expand the number of published solutions and utilize them as part of their own mission that aligns with the goals of PANORAMA. Other considerations include the potential dissolvement of the platform's portal structure, as thematic overlaps between portals will increase.

PANORAMA at present is a partnership of 5 institutions, with active interest from further partners to join in and invest in it. The added value to these partners is that a strong network, "brand recognition", powerful web tool, and knowledge management methodology has already been developed and is ready to be used by these institutions – almost like a "module" which can be plugged into projects to e.g. meet knowledge management requirements. Interest of new institutions, as well as further units within the existing partners, to use PANORAMA, will only increase the more known the initiative becomes,

in turn enhancing its sustainability, and adding to the ongoing efforts of the existing partners to ensure continuous funding beyond the life span of the projects that helped to establish PANORAMA

Given that PANORAMA covers diverse PA solutions that are not limited by geography or topic, with new solutions continuously being added, PA stakeholders around the globe can look to this platform as an efficient means of accessing and sharing knowledge on PA management. IUCN and GIZ, along with the other partners, are actively identifying relevant related initiatives and platforms, and encourage solution providers to contribute to these as well where appropriate. An example collaboration involved university scientists working on a project examining challenges and solutions for protected area managers in transboundary areas, and through this joint effort 9 solutions on transboundary conservation were published (Rodrigues and Fischborn 2016). In the future, similar collaborations will be pursued. Working in parallel, related initiatives can display whole or parts of relevant PANORAMA solutions on their case-study web portals to serve their more targeted user communities. Such collaboration is another mechanism to expand the diversity of partners and user base for PANORAMA.

References

Downie, B. 2017. Improving relationships between local communities and Saadani National Park management. In: PANORAMA - Solutions for a Healthy Planet. Retrieved, from http://panorama.solutions/en/solutions/improving-relationships-between-local-communities-and-saadani-np-management

Downie, B. K. 2015. Conservation influences on livelihood decision-making: a case study from Saadani National Park, Tanzania. PhD Thesis. Retrieved, from https://dspace.library.uvic.ca/handle/1828/6266

Downie, B. K. 2016. Workshops in Saadani National Park with Linking Tourism & Conservation for community centered learning from protected area tourism solutions. Retrieved, from http://www.thekeshotrust.org/cms/wp-content/images/IUCN-final-report.pdf

IUCN. 2015. Natural solutions. Retrieved 28 February 2018, from https://www.iucn.org/theme/protected-areas/publications/natural-solutions

Kesho Trust. 2017. Promoting Environmental Conservation and Cooperation. Retrieved, from http://www.thekeshotrust.org/projects/pecc/

Kujirakwinja, D. 2017. Conflict resolution strategy for Kahuzi-Biega National Park. In: PANORAMA - Solutions for a Healthy Planet. Retrieved, from http://panorama.solutions/en/solutions/conflict-resolution-strategy-for-kahuzi-biega-national-park

Rodrigues, D. C., and M. Fischborn. 2016. Solutions in focus : transboundary protected area solutions. Retrieved 8 November 2017, from https://portals.iucn.org/library/node/46613

Snyman, S. 2017. Making protected area concessions work for communities. In: PANORAMA - Solutions for a Healthy Planet. Retrieved, from http://panorama.solutions/en/content/making-protected-area-concessions-work-communities
